# Comparative proteomic profiling of plasma exosomes in lung cancer cases of liver and brain metastasis

**DOI:** 10.1186/s13578-023-01112-5

**Published:** 2023-09-28

**Authors:** Sini Li, Yan Qu, Lihui Liu, Xue Zhang, Yan He, Chao Wang, Yufeng Guo, Li Yuan, Zixiao Ma, Hua Bai, Jie Wang

**Affiliations:** 1 Zhejiang Cancer Hospital, Hangzhou Institute of Medicine (HIM), Chinese Academy of Sciences, Hangzhou, 310022 Zhejiang China; 2 CAMS Key Laboratory of Translational Research On Lung Cancer, State Key Laboratory of Molecular Oncology, Department of Medical Oncology, National Cancer Center/National Clinical Research Center for Cancer/Cancer Hospital, Chinese Academy of Medical Sciences and Peking Union Medical College, Beijing, 100021 China; 3 National Cancer Center/National Clinical Research Center for Cancer/Cancer Hospital, Chinese Academy of Medical Sciences and Peking Union Medical College, Beijing, 100021 China; 4 Department of Radiotherapy, Shandong Provincial Hospital Affiliated to Shandong First Medical University, Jinan, 250021 Shandong China

**Keywords:** Lung cancer, Liver metastasis, Brain metastasis, Proteomics, Exosomes, Biomarkers

## Abstract

**Background:**

Metastases within liver or the brain are the most common causes of mortality from lung cancer (LC). Predicting liver or brain metastases before having evidence from imaging of the tumors is challenging but important for early patient intervention. According to mounting evidence, exosomes circulating within blood may facilitate cancer spread by transporting certain proteins for target cells.

**Methods:**

Using liquid chromatography–MS/MS, we investigated the plasma exosomes’ proteomic profiles derived from 42 metastatic LC patients [16 solitary liver metastasis (LM), together with 26 solitary brain metastasis (BM)] and 25 local advanced (LA) lung cancer cases without metastasis, together with five healthy controls (HC), assessing the LM and BM pathogenesis and find potential novel organ-designated proteomic biomarkers. Using ELISA assay, we verified the expression levels of three plasma exosomal protein biomarkers in 110 LC patients, including 40 solitary LM, 32 solitary BM and 38 LA, and 25 HC.

**Results:**

In total, 143 and 120 differentially expressed exosome-based proteins (DEEPs) were found to be dysregulated in LM and BM of lung cancer (LM-DEEPs, BM-DEEPs), compared for LA lung cancer samples, respectively. The bioinformatics analyses indicated the heterogeneity and homogeneity in LM-DEEPs and BM-DEEPs. They were primarily engaged within proteomic triggering cascade, ECM-receptor interaction, and the collagen-containing extracellular matrix. Regarding heterogeneity, LM-DEEPs primarily consisted of proteoglycans, lipoprotein, integrin, and heat shock protein, whereas the BM-DEEPs consisted of calcium-dependent/S100 proteins. Furthermore, small cell lung cancer (SCLC) and non-small cell lung cancer (NSCLC)-plasma-stemming exosome proteomics showed heterogeneity, which helped to explain some of the differences between SCLC and NSCLC's metastatic features. We also found that SELL and MUC5B could be used as diagnostic markers of BM, while APOH, CD81, and CCT5 could help diagnose LM in LC patients. Additionally, we demonstrated in a validation cohort that MUC5B and SELL could serve as biomarkers for diagnosing BM, and APOH could be a novel potential diagnostic biomarker of LM.

**Conclusion:**

We presented the comprehensive and comparative plasma-stemming exosomes’ proteomic profiles from cases of LC who had isolated liver and brain metastases for the first time. We also suggested several possible biomarkers and pathogenic pathways that might be a great starting point for future research on LC metastasis.

**Supplementary Information:**

The online version contains supplementary material available at 10.1186/s13578-023-01112-5.

## Background

The primary factor in lung cancer (LC) related fatalities is distant metastasis, an unavoidable lung cancer growth outcome [1]. Most lung cancers metastasize within brain (15–43%) and liver (33–40%), and various clinical lung cancer subtypes have specific favored metastatic locations [2]. In particular, brain metastasis (BM) and liver metastasis (LM) are more prevalent in SCLC patients, while BM is more prevalent in NSCLC patients [3, 4]. The intricacy of the precise molecular process mechanism causing the metastasis makes it difficult to accurately diagnose and treat LC with LM or BM [5, 6]. To comprehend the pathophysiology, enhance diagnosis, and offer efficient, customized therapy solutions for LC cases having LM and BM, innovative biomarkers with high specificity and sensitivity are required. In recent years, the discovery of a new mode of exosome-mediated intercellular communication at the local and secondary metastatic sites has provided fresh insights into tumor metastasis mechanisms and potential therapeutic targets for treating different types of cancers [7–9].

Exosomes are spherical or cup-shaped, of endosomal origin, and have a diameter between 30 and 150 nm. Cells produce extracellular vesicles that are widely distributed in physiological fluids, including blood, saliva, and urine [10]. Within pathophysiological state, almost all cells secrete exosomes carrying different functional molecules, including proteins, lipids, and nucleic acids, which regulate several biological and pathological processes such as inflammation and immune response, tumor growth, and metastasis [11, 12]. The tumor microenvironment remodeling and formation made it more favorable for metastasis and allowed the discovery of cancer diagnostic biomarkers as well as for the prognosis and therapeutic response prediction of metastatic cancer patients, which have all been shown to be significantly influenced by exosomes, according to a growing body of research [13, 14]. Additionally, because of their great affinity for certain target cells, the exosomes carried proteins are thought to play a significant role in organ-specific metastasis [8, 15, 16]. For instance, exosome-based ITGαVβ5 promotes liver metastasis, whereas ITGβ3 promotes brain tropic metastasis [15]. Plasma exosomes from LC cases having LM or BM have not yet been subjected to proteome studies.

In this investigation, to assess the pathophysiology of liver and brain metastasis and identify possible new organ-specific proteome biomarkers, plasma-stemming exosomes from cases of metastatic LC (LM and BM), healthy control (HC), and local advanced (LA) lung cancer cases without metastasis were subjected to comparative proteomics profiling analysis. This investigation demonstrated the heterogeneity and homogeneity in exosome-based proteomics between liver and brain metastasis, which may aid in explaining organotropic metastasis for liver or brain in cases of lung cancer. Additionally, there were changes within plasma-stemming exosome-based proteome profiles from SCLC and NSCLC patients who had liver and brain metastases, which helps to partly elucidate the diverse metastatic features of SCLC and NSCLC patients. Finally, we assessed the diagnostic potential of several biomarkers for foretelling liver and brain metastases in cases of LC and validated their diagnostic value in a validation cohort. Future studies on LM and BM in LC should find great value in our findings.

## Results

### Characterization of the plasma-stemming exosomes

**Fig. 1:**
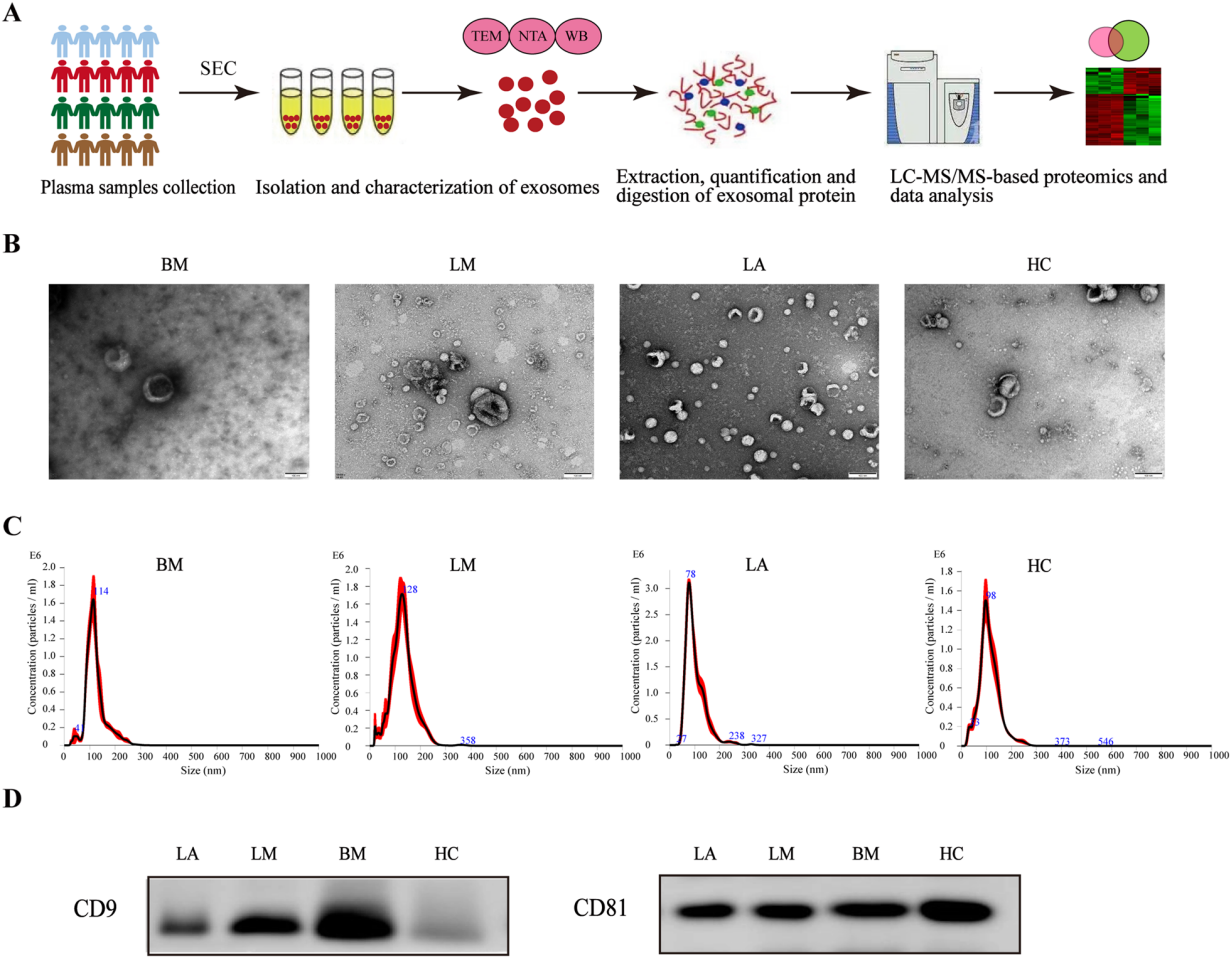
Characterization of the plasma-stemming exosomes. **A** Experimental workflow showed the isolation and proteomic analyses of plasma-stemming exosomes. **B** TEM showed the shape of plasma-stemming exosomes. The bar indicates 100 nm. **C** NTA showed the size distribution of plasma-stemming exosomes. **D** WB detected the exosome-based surface marker proteins CD9 and CD81

In Fig. 1A, the overall experimental layout is shown. Size exclusion chromatography (SEC) was employed to separate exosomes from the plasma samples, and transmission electron microscope (TEM), nanoparticle tracking analysis (NTA), and WB then confirmed them. The plasma-stemming exosomes were shown by TEM examination to be typical cup-shaped vesicles (Fig. 1B). The isolated particles were shown to have a size range between 30 and 150 nm by NTA (Fig. 1C). By using WB, we confirmed the recognizable exosome-based surface markers’ presence such as CD9 and CD81 (Fig. 1D). Together, the data showed that it was possible to identify and purify plasma-stemming exosomes, setting the groundwork for later proteomic investigations.

### Proteomic profiling for plasma-stemming exosomes

**Fig. 2:**
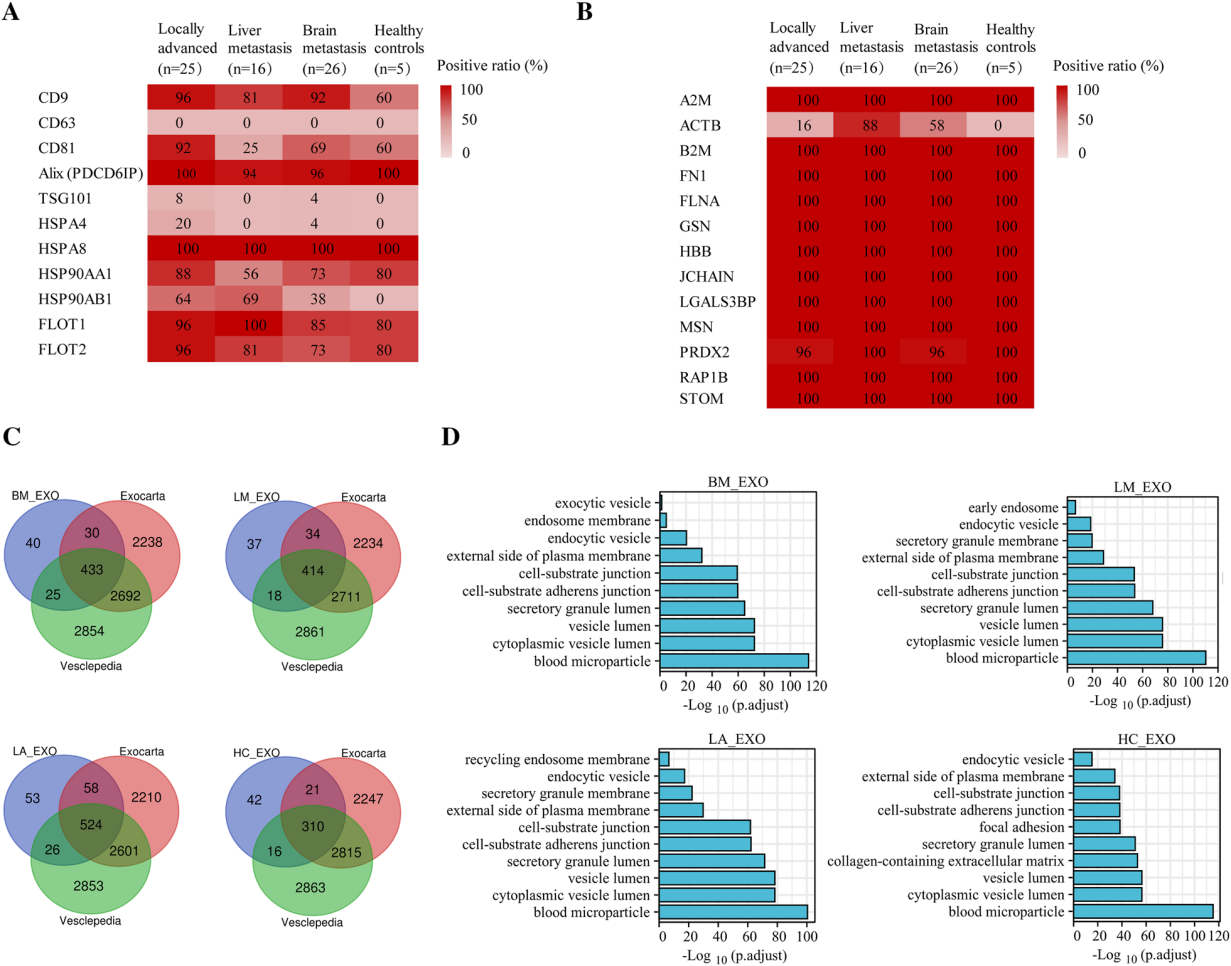
Proteomic profiling for plasma-stemming exosomes. **A**, **B** Positivity for 11 standard and 13 newly described exosome-based protein markers in four cohorts, respectively. Each box is labeled with the positive rate (%) of the specified protein in each cohort. Dark red represents higher positive rate. **C** Venn diagram displayed the exosome-based proteins identified in this investigation compared to documented vesicular proteins within Exocarta/Vesiclepedia repositories. **D** The cellular component (CC) of GO analysis of plasma-stemming exosomes

Quantitative LC–MS/MS proteomics found 1482 exosome-based proteins across all four cohorts (Additional file 1: Table S1). We looked at the expressions of well-known exosome-based protein makers to define the exosomes [17]. Alix, CD9, HSP90AA1, HSPA8, FLOT1, and FLOT2 were discovered in more than 50% of the samples from the four cohorts out of the 11 standard exosome-based markers; however, CD63 was absent in our samples (Fig. 2A). Except for actin beta (ACTB), all 13 of the newly described exosome-based markers were found. The aforementioned findings supported the exosomes' purity (Fig. 2B). We filtered the discovered exosome-based proteins to reveal the exosomes’ proteomic profiles from the four cohorts, and those with more than 30% LFQ intensity ≠ 0 in each cohort were maintained for further investigation. As a result, 503, 528, 389, and 661 proteins were recognized within LM, BM, HC, and LA cohorts, respectively. When we compared them for documented vesicular proteins within Exocarta [18, 19]/Vesiclepedia [20] repositories, this investigation identified more than 80% were common (Fig. 2C). Moreover, 60% to 80% from top-ranking 100 were found within four groups (Additional file 2: Figure S1A), demonstrating significant exosome-based enrichment within exosome library. The discovered proteins were considerably enriched within cytoplasmic areas and extracellular exosome category constituents, according to Gene Ontology (GO) analysis’s cellular component (CC) (Fig. 2D). Additionally, KEGG pathway analysis revealed the more substantial abundance of these proteins in endocytosis (Additional file 3: Figure S2). According for aforementioned information, SEC-isolated exosomes were loaded with exosome-based proteins.

### Determination/assessment for role relevance enrichment within brain metastases-linked differentially expressed exosome-based proteins (BM-DEEPs)

**Fig. 3:**
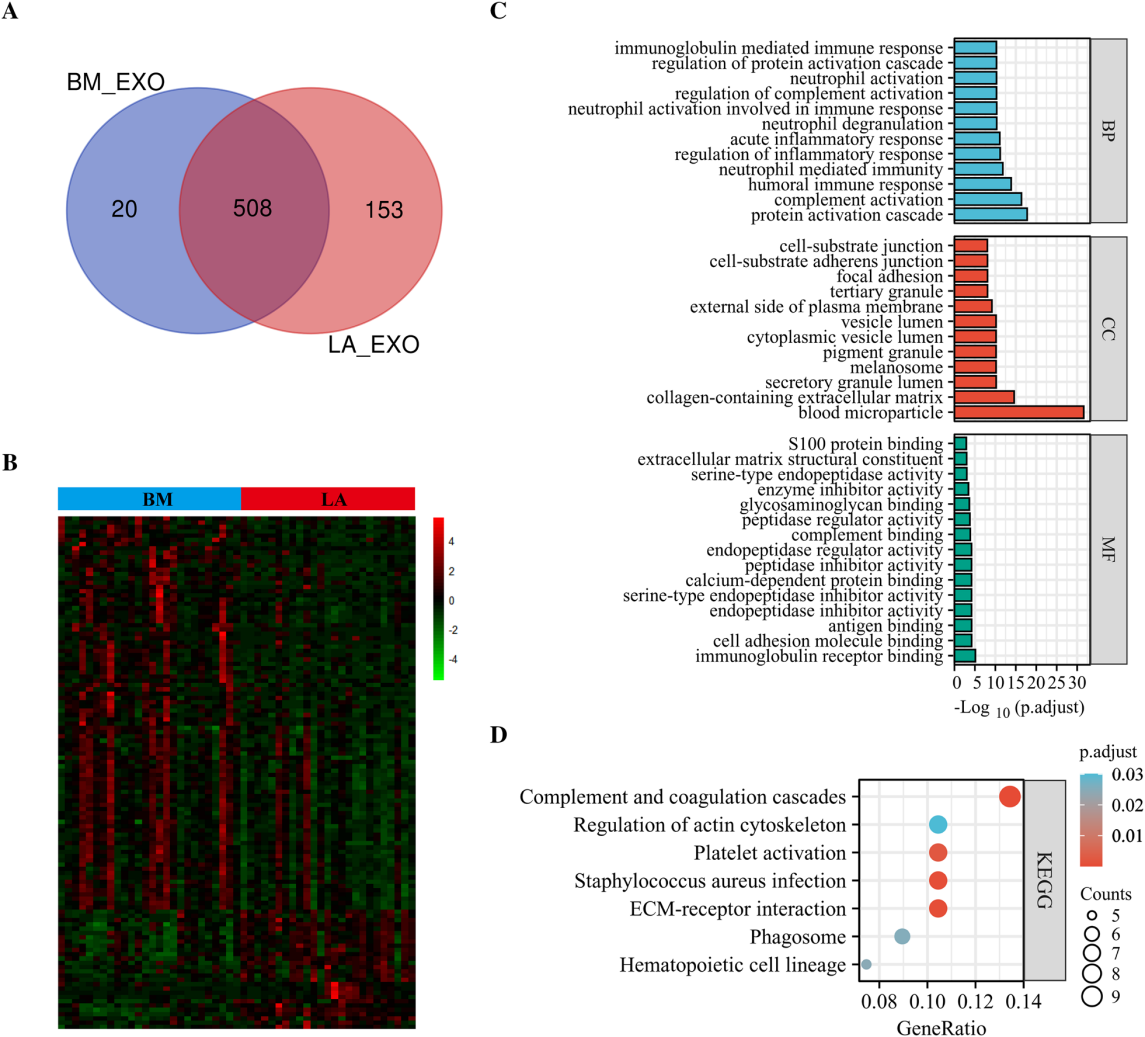
Bioinformatics analysis of brain metastases-linked differentially expressed exosome-based proteins (BM-DEEPs). **A** Comparison between exosomal proteins from BM and LA patients. **B** Heatmap of 120 BM-DEEPs between BM and LA patients. **C**, **D** GO annotations and KEGG analysis of the BM-DEEPs

**Fig. 4:**
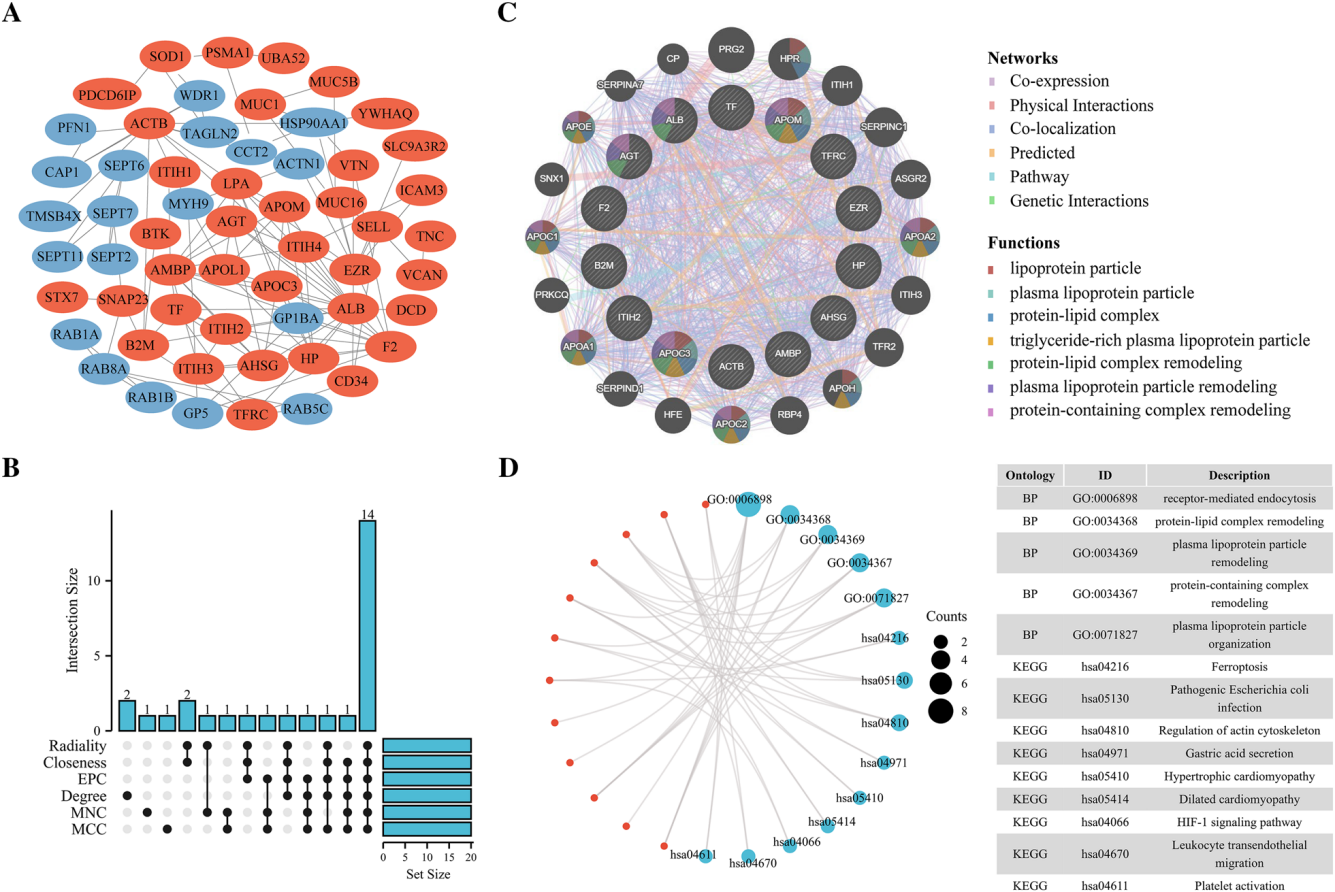
Bioinformatics analysis of brain metastases-linked differentially expressed exosome-based proteins (BM-DEEPs). **A** Protein‑protein interaction (PPI) network of the BM-DEEPs. Red and blue plots indicated upregulated and downregulated BM-DEEPs, respectively. **B** Venn diagram showed that 14 overlapping hub BM-DEEPs were obtained from “cytoHubba” plugin. **C** 14 hub overlapping BM-DEEPs were analyzed by GeneMANIA database. **D** GO annotations and KEGG analysis of 14 overlapping hub BM-DEEPs

We examined exosomes using a Venn diagram to determine differing proteins expressed within LA/BM cohorts. This identified 508 common proteins, whereas 153 and 20 were exclusive to LA and BM, respectively (Fig. 3A). Then, using the criterion of log2 |fold-change|≥ 1 together with p < 0.05, BM-DEEPs were determined. 120 BM-DEEPs, comprising 28 downregulated and 92 upregulated proteins, were found, as revealed by the heat map (Fig. 3B). There is a list of the BM-DEEPs in Additional file 1: Table S2. We carried out KEGG pathway and GO enrichment studies to assess role relevance for BM-DEEPs. Molecular function (MF), cellular component (CC), and biological process (BP) were three groups of findings from BM-DEEPs’ GO enrichment assessment. Regarding the BP, complement activation and proteomic triggering cascade were two primary functions of the BM-DEEPs. Regarding the MF, antigen binding, immunoglobulin receptor binding enrichment, and regarding CC, blood microparticle enrichment of BM-DEEPs was observed (Fig. 3C). KEGG assessment highlighted coagulation and complement cascades were abundant within BM-DEEPs (Fig. 3D). BM-DEEPs’ interactions were then clearly depicted in a complete network that we built utilizing the STRING database to study the relationships between the BM-DEEPs (Fig. 4A). Afterward, the best 20 hub BM-DEEPs were selected using the 6 available cytoHubba plug-in algorithms. We found 14 common hub proteins by taking the Venn diagrams intersection, including AMBP, ALB, F2, AHSG, ITIH2, APOM, TF, HP, ACTB, AGT, EZR, B2M, APOC3 and TFRC (Fig. 4B). We also examined the aforementioned proteins using the GeneMANIA database. These proteins displayed an intricate PPI networkings with physical interactions of 21.01%, co-expression of 55.15%, co-localization of 12.81%, predicted of 5.43%, and pathway of 5.28%. (Fig. 4C). According for GO analysis, 14 overlapping hub proteins were mostly engaged for modifying protein-lipid complexes and receptor-mediated endocytosis (Fig. 4D). Ferroptosis, HIF-1 signaling, actin cytoskeleton regulation, and leukocyte trans-endothelial migration were the most heavily enriched pathways within KEGG study (Fig. 4D).

### Determination/assessment for role relevance profiles within liver metastases-linked differentially expressed exosome-based proteins (LM-DEEPs)

**Fig. 5:**
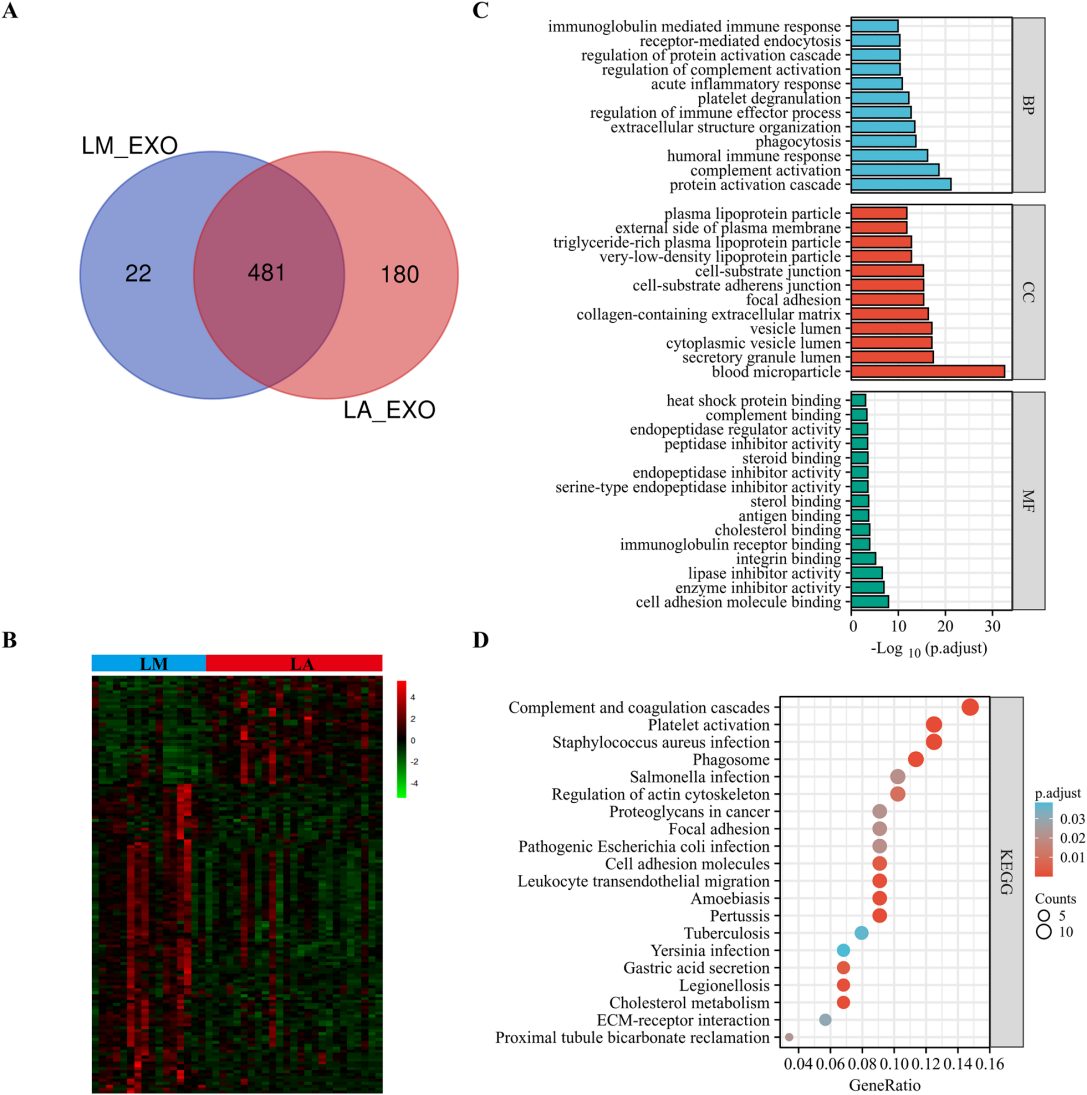
Bioinformatics analysis of liver metastases-linked differentially expressed exosome-based proteins (LM-DEEPs). **A** Comparison between exosomal proteins from LM and LA patients. **B** Heatmap of 143 LM-DEEPs between LM and LA patients. **C**, **D** GO annotations and KEGG analysis of the LM-DEEPs

**Fig. 6:**
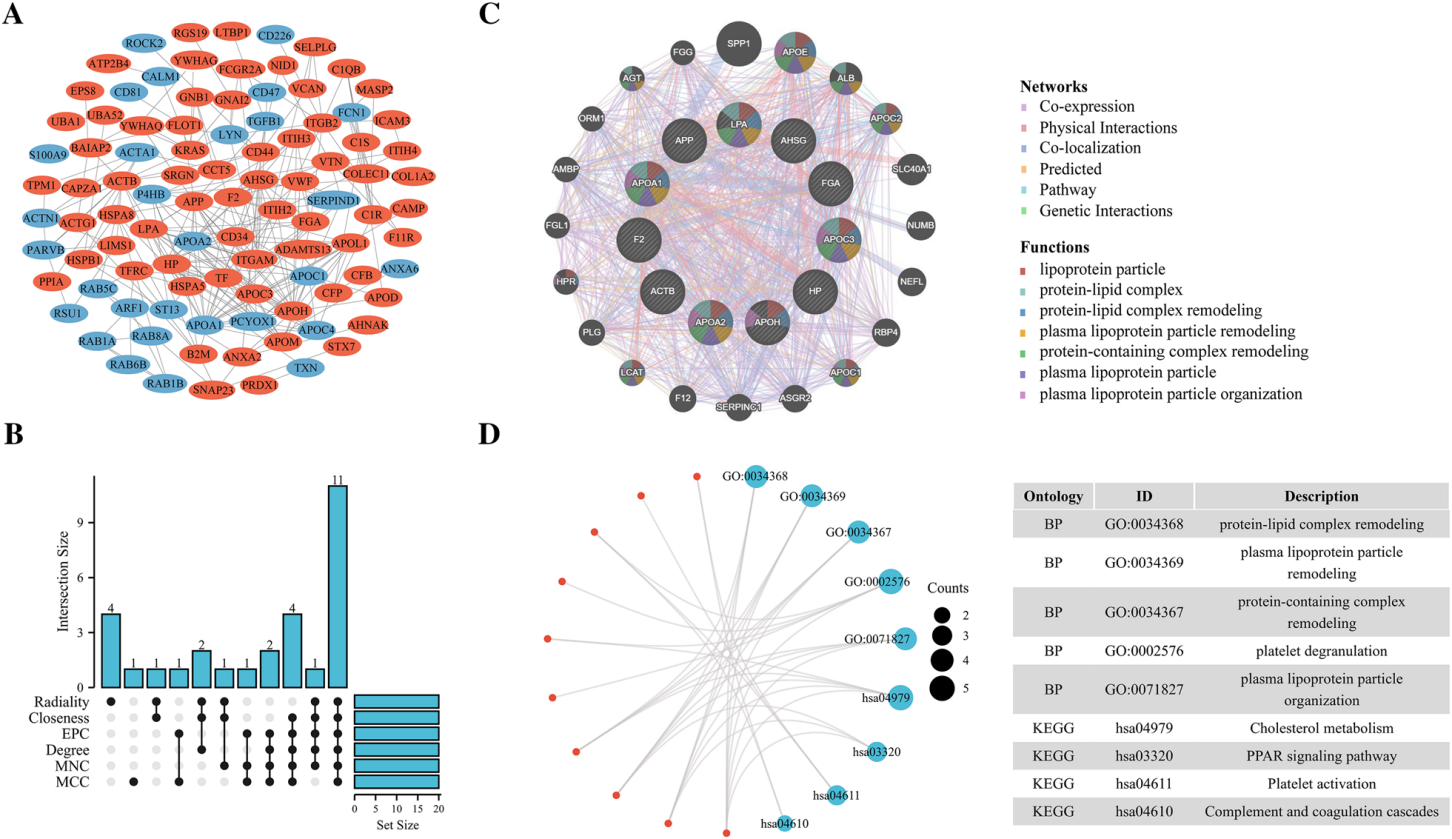
Bioinformatics analysis of liver metastases-linked differentially expressed exosome-based proteins (LM-DEEPs). **A** Protein‑protein interaction (PPI) network of the LM-DEEPs. Red and blue plots indicated upregulated and downregulated LM-DEEPs, respectively. **B** Venn diagram showed that 11 overlapping hub LM-DEEPs were obtained from “cytoHubba” plugin. **C** 11 hub overlapping LM-DEEPs were analyzed by GeneMANIA database. **D** GO annotations and KEGG analysis of 11 overlapping hub LM-DEEPs

Using bioinformatics methods, we screened and compared LM-DEEPs between the LM and LA groups to see the exosome-based proteins landscape between LM and BM of LC patients, which was previously unknown. The exosome-based proteins found within LM and LA that are both common and distinctive were shown within Venn diagram. The two cohorts shared a total of 481 proteins, while 22 proteins (LM) and 180 proteins (LA) were uniquely expressed (Fig. 5A). The LM-DEEPs were then chosen through log2 |fold-change|≥ 1 together with p < 0.05. Overall, 143 LM-DEEPs were found, comprising 37 downregulated and 106 upregulated proteins, as revealed by the heat map (Fig. 5B). In Additional file 1: Table S3, the findings of the LM-DEEPs are presented in detail. We also performed KEGG and GO studies to investigate the LM-DEEPs’ functional characteristics. Figure 5C presents the findings of the GO analysis. Interestingly, LM-DEEPs were highly abundant for proteomic triggering cascade and blood microparticles, respectively, regarding the BP and CC, which were similar for BM-DEEPs. As with the MF, the LM-DEEPs exhibited improved binding to integrin, cholesterol, heat shock protein, and cell adhesion molecules. Utilizing KEGG pathway annotations, LM-DEEPs were found to be particularly rich for pathways related to cholesterol metabolism, leukocyte trans-endothelial migration, cancer proteoglycans, and complement and coagulation cascades (Fig. 5D). The LM-DEEPs’ relationship is seen in Fig. 6A. Additionally, we discovered 11 hub proteins (APOA1, APOH, APOC3, APOA2, APP, HP, AHSG, LPA, FGA, F2, ACTB) that were recognized by all six methods (Fig. 6B). According for GeneMANIA database, the aforementioned proteins displayed an intricate PPI networking having physical interactions of 25.74%, co-expression of 46.68%, predicted of 1.17%, co-localization of 22.98%, and pathway of 3.14% (Fig. 6C). According to GO analysis, such 11 hub LM-DEEPs were mostly engaged for modifying protein-lipid complexes (Fig. 6D). Additionally, KEGG assessment highlighted aforementioned hub LM-DEEPs were abundant within PPAR signaling pathway and cholesterol metabolism (Fig. 6D).

### Proteomic profiles of plasma-stemming exosomes from SCLC and NSCLC patients with BM and LM exhibit different landscapes

**Fig. 7:**
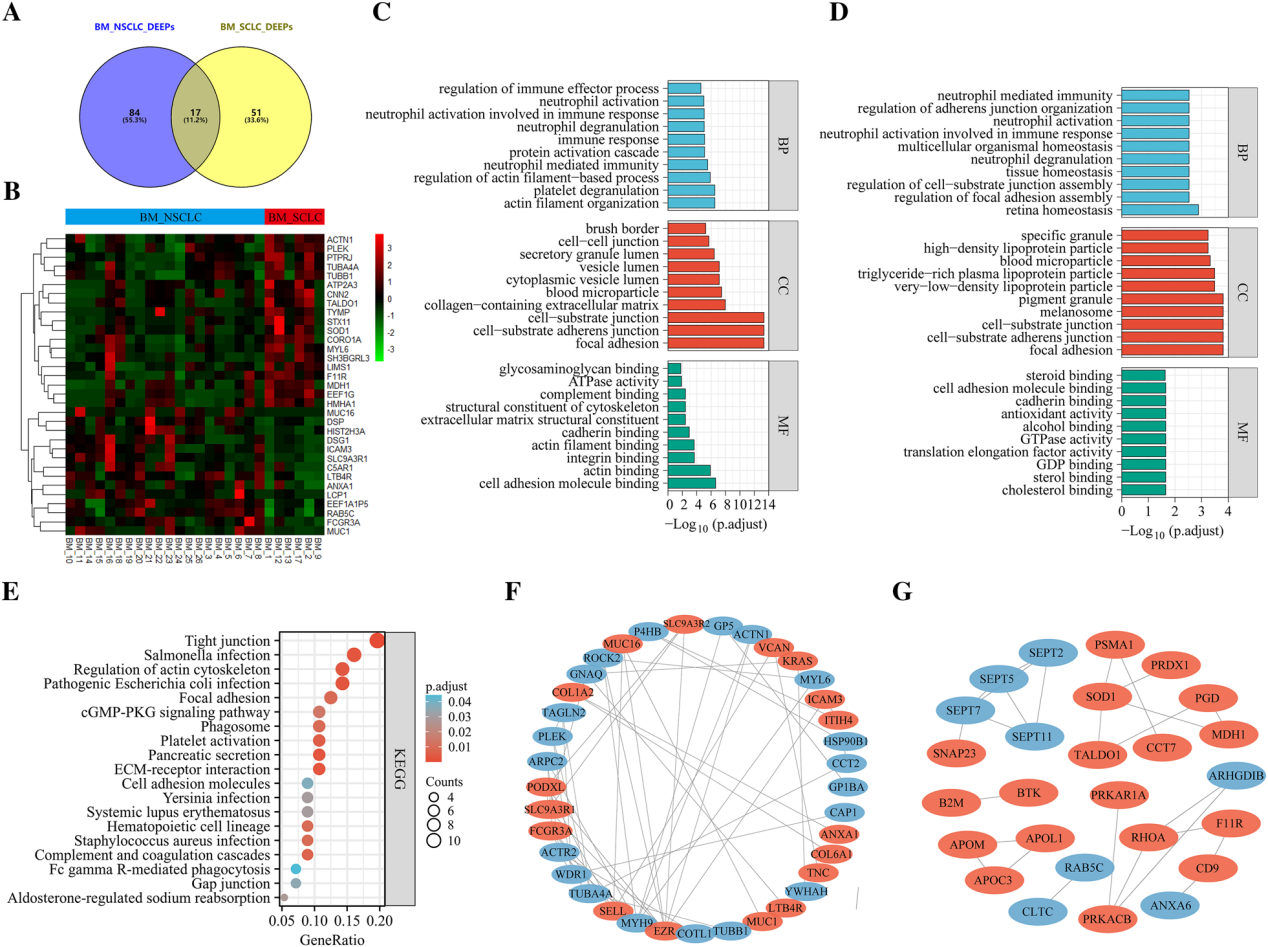
Bioinformatics analysis of differentially expressed exosome-based proteins associated with BM in NSCLC and SCLC (BM_NSCLC_DEEPs and BM_SCLC_DEEPs). **A** Comparison between BM_NSCLC_DEEPs and BM_SCLC_DEEPs. **B** Heatmap indicated a few distinctive proteins expressed differently in BM patients in SCLC and NSCLC. **C**, **D** GO annotation analysis of the specific BM_NSCLC_DEEPs and BM_SCLC_DEEPs, respectively. **E** KEGG pathway analysis of the specific BM_NSCLC_DEEPs. **F**, **G** Protein‑protein interaction (PPI) network of the specific BM_NSCLC_DEEPs and BM_SCLC_DEEPs, respectively. Red and blue plots indicated upregulated and downregulated proteins, respectively

After classifying cases of LA and BM into SCLC and NSCLC, differential analysis was performed to distinguish differentially expressed exosome-based proteins associated with BM in NSCLC and SCLC (BM_NSCLC_DEEPs and BM_SCLC_DEEPs). 51 and 84 proteins, respectively, were specific for BM_SCLC_DEEPs/BM_NSCLC_DEEPs (Fig. 7A). Additionally, heat-mapping indicated a few distinctive proteins expressed differently in BM patients in SCLC and NSCLC (Fig. 7B). In Fig. 7C and D, the findings from the GO analysis are shown. The results showed that the BM-DEEPs in SCLC and NSCLC each have distinctive properties. For instance, regarding BP, the distinctive BM_NSCLC_DEEPs were abundant within actin filament organization/platelet degranulation, while the distinctive BM_SCLC_DEEPs were primarily engaged within cell-substrate junction assembly and focal adhesion assembly. The distinctive BM_NSCLC_DEEPs were considerably abundant within tight junction/actin cytoskeleton control within KEGG study (Fig. 7E). The distinctive BM_SCLC_DEEPs, however, did not possess important routes. Figure 7F and G show that this investigation advanced the development of PPI networking, concerning BM_NSCLC_DEEPs and BM_SCLC_DEEPs.

**Fig. 8:**
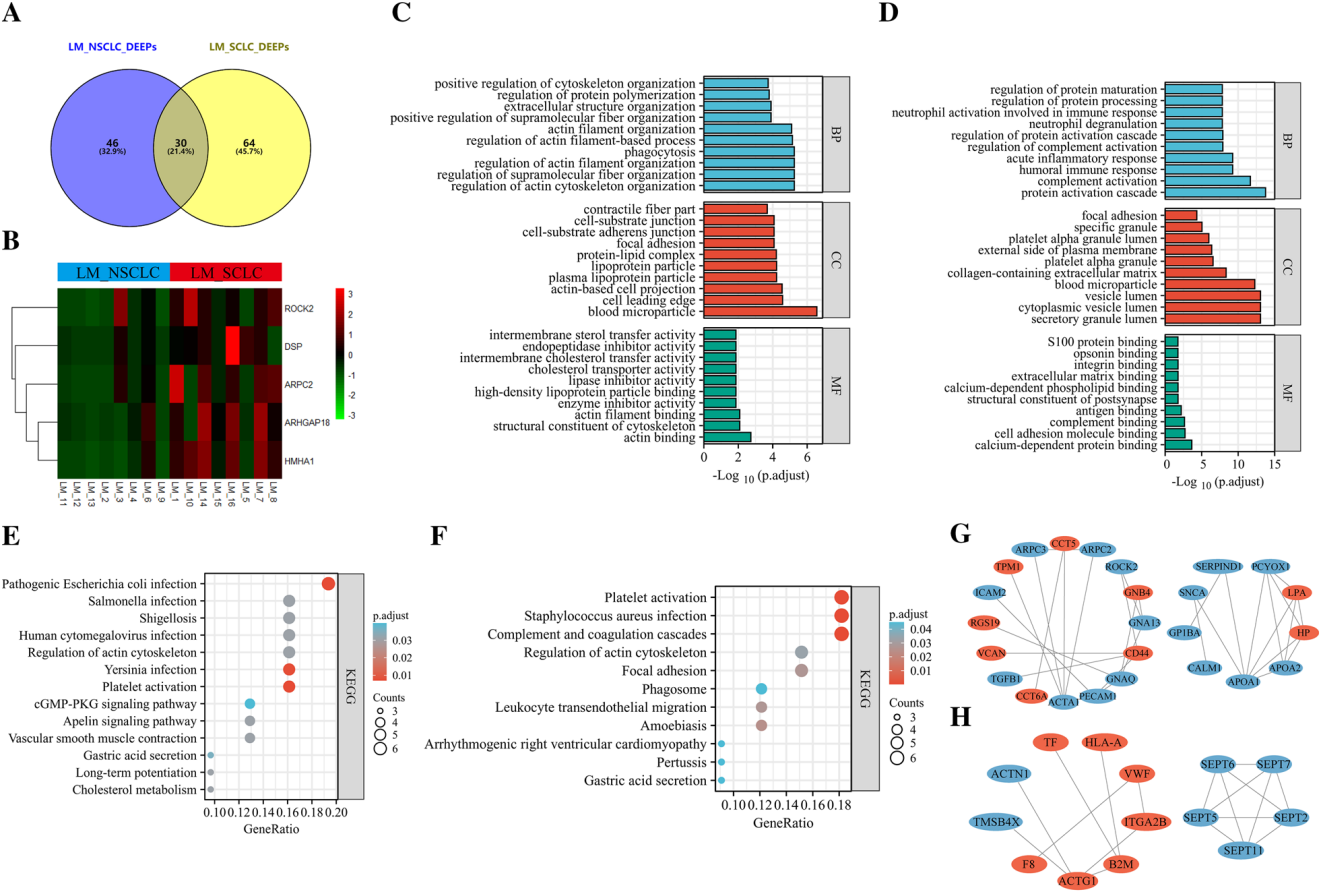
Bioinformatics analysis of differentially expressed exosome-based proteins associated with LM in NSCLC and SCLC (LM_NSCLC_DEEPs and LM_SCLC_DEEPs). **A** Comparison between LM_NSCLC_DEEPs and LM_SCLC_DEEPs. **B** Heatmap indicated a few distinctive proteins expressed differently in LM patients in SCLC and NSCLC. **C**, **D** GO annotation analysis of the specific LM_NSCLC_DEEPs and LM_SCLC_DEEPs, respectively. **E**, **F** KEGG pathway analysis of the specific LM_NSCLC_DEEPs and LM_SCLC_DEEPs, respectively. **G**, **H** Protein‑protein interaction (PPI) network of the specific LM_NSCLC_DEEPs and LM_SCLC_DEEPs, respectively. Red and blue plots indicated upregulated and downregulated proteins, respectively

The DEEPs within LM from cases of SCLC and NSCLC were also obtained using the same technique (LM_SCLC_DEEPs and LM_NSCLC_DEEPs, respectively). 64 and 46 proteins, respectively, were specific to LM_SCLC_DEEPs and LM_NSCLC_DEEPs, as shown in Fig. 8A. The heat map demonstrated many distinctive proteins that were differently expressed in SCLC and NSCLC cases of LM (Fig. 8B). The distinctive LM_NSCLC_DEEPs were primarily engaged within regulating supramolecular fiber organization and actin cytoskeleton organization. In contrast, the distinctive LM_SCLC_DEEPs were primarily abundant within complement and proteomic triggering cascade, following GO assessment for distinctive LM_NSCLC_DEEPs/LM_SCLC_DEEPs (Fig. 8C and D). The distinctive LM_NSCLC_DEEPs demonstrated to be implicated within cholesterol metabolism and apelin signaling pathway by KEGG pathway analysis (Fig. 8E). At the same time, the LM_SCLC_DEEPs were found to be engaged within complement/coagulation cascades and actin cytoskeleton control (Fig. 8F). Additionally, Fig. 8G and H show PPI networks for distinctive LM_NSCLC_DEEPs/LM_SCLC_DEEPs. Summarizing, the data above showed that plasma-stemming exosome-based proteins from SCLC/NSCLC patients were heterogeneous.

### Diagnostic utility for plasma-stemming exosome-based proteins in lung cancer metastases (hepatic/cerebral)

**Fig. 9:**
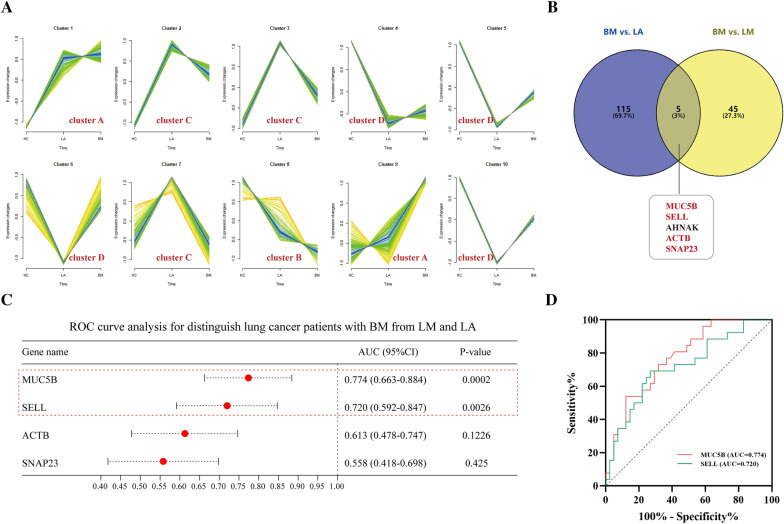
Diagnostic utility for the selected plasma-stemming exosome-based proteins in lung cancer patients with BM. **A** 10 soft clusters of BM. The horizontal axis indicates the initiation and progression of BM (HC, LA, and BM). The vertical axis indicates the expression of exosome-based proteins. **B** Nested analyses of BM versus LA and BM versus LM. ACTB, MUC5B, SNAP23, and SELL were in cluster A. **C**) ROC curves assessed diagnoses capability for the four BM-distinct proteins. **D** The area under the ROC curve of MUC5B and SELL

Two screening techniques were used to identify the important DEEPs with diagnostic potential. First, we investigated the patterns within exosome-based protein expression within HC, LA, and LM or BM cohorts using the soft clustering approach. Mfuzz analysis produced a total of 10 clusters. We divided these 10 clusters into major classes using the soft clustering of BM, including clusters A (clusters 1 and 9), B (cluster 8), C (clusters 2, 3, and 7), and cluster D (clusters 4, 5, 6 and 10) (Additional file 1: Table S4). As BM developed, the cluster A proteomic expression was upregulated, whereas cluster B proteins followed an opposing pattern (Fig. 9A). Consequently, this investigation concluded that cluster A and cluster B's total exosome-based proteins were linked to BM. Additionally, we identified 50 DEEPs by contrasting the exosome-based proteins in LM and BM (Additional file 1: Table S5). Five proteins were shown to be related explicitly to BM in further nested analyses (LA versus BM and LM versus BM). These proteins are ACTB, MUC5B, SNAP23, SELL, and AHNAK (Fig. 9B). These 5 distinct proteins were compared to proteins in clusters A and B, and it was discovered that ACTB, MUC5B, SNAP23, and SELL were in cluster A, suggesting that they may have been involved in initiation and progression of BM. Receiver operating characteristic (ROC) curves assessed diagnoses capability for aforementioned four proteins (Fig. 9C). Only MUC5B and SELL showed some accuracy within BM diagnosis, according to ROC curves (AUC = 0.774, specificity = 0.732, sensitivity = 0.692; AUC = 0.720, specificity = 0.732, sensitivity = 0.692) (Fig. 9C and D). The false positive rate and false negative rate of MUC5B and SELL were 26.8% and 30.8%, respectively, indicating their potential use as biomarkers for BM detection in cases of LC. We also investigated exosome-based proteins in diagnosing BM within NSCLC/SCLC patients using the two screening techniques indicated above (Additional file 4: Figure S3A, B and Additional file 5: Figure S4A, B). Nested analyses between NSCLC_BM and NSCLC_LA and NSCLC_BM and NSCLC_LM identified 22 proteins specific to BM in NSCLC, as shown in Additional file 4: Figure S3B. SELL, ACTB, DSG1, and PODXL were in cluster A, suggesting they are tightly connected with BM in NSCLC, which was discovered by comparing these 22 distinct proteins with those in clusters A and B. ROC curves suggested that SELL, DSG1 and PODXL proteins might be used as biomarkers for BM detection in NSCLC patients, and the trio together had a higher diagnostic value (AUC = 0.844) (Additional file 4: Figure S3C, D). However, with an AUC of 0.889, only CD9 was shown to have a diagnostic potential for BM detection in SCLC (Additional file 5: Figure S4C).

**Fig. 10:**
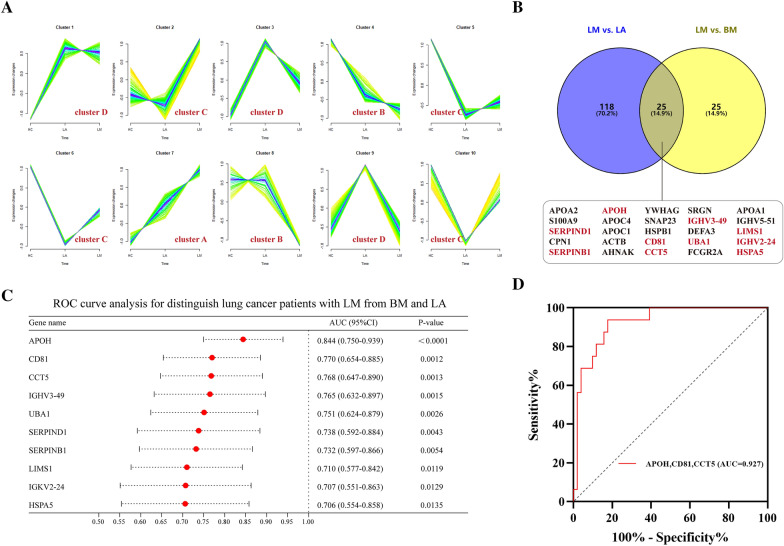
Diagnostic utility for the selected plasma-stemming exosome-based proteins in lung cancer patients with LM. **A** 10 soft clusters of LM. The horizontal axis indicates the initiation and progression of LM (HC, LA, and LM). The vertical axis indicates the expression of exosome-based proteins. **B** Nested analyses of LM versus LA and BM versus LM. 10 proteins belonged to the overlapping proteins of LM versus LA and BM versus LM, as well as to cluster A or B proteins. **C** ROC curves assessed the diagnoses capability for the 10 LM-distinct proteins. **D** The top three exosomal proteins combinations had the greatest AUC (0.927) for distinguishing between LM and LA, and BM patients

Additionally, we looked at the diagnostic indicators linked to LM in cases of LC. The 10 clusters of LM exosome-based proteins were broken down into (Additional file 1: Table S6); cluster A (which includes cluster 7), cluster B (which includes clusters 4 and 8), cluster C (which includes clusters 2, 5, 6, and 10), together with cluster D (which includes clusters 1, 3 and 9). Cluster A proteins saw an upregulation among them with the development of LM. However, the cluster B proteins experienced an opposite trend in their expression (Fig. 10A). After that, nest-based comparative analyses (LA versus LM and LM versus BM) revealed LM to solely have 25 DEEPs (Fig. 10B). These 25 distinct proteins were compared to exosome-based proteins from clusters A and B, and it was discovered that CCT5, APOH, SERPINB1, UBA1, HSPA5, and LIMS1 belonged for former, while IGHV3-49, CD81, IGKV2-24, and SERPIND1 belonged for latter. The next step was to create ROC curves to assess the ability of the 10 exosome-based proteins to diagnose LM (Fig. 10C). APOH (AUC = 0.844; specificity = 0.804; sensitivity = 0.813); CD81 (AUC = 0.770; specificity = 0.529; sensitivity = 1.000); and CCT5 (AUC = 0.768; specificity = 0.745; sensitivity = 0.750) were the best classifiers. The false positive rates of APOH, CD81 and CCT5 were 19.6%, 47.1% and 25.5%, and the false negative rates were 18.7%, 0.0% and 25.0%, respectively. Interestingly, Fig. 10D reveals that they had the greatest AUC (0.927) for distinguishing between LM and LA, and BM patients. Diagnoses-linked indicators for LM within NSCLC/SCLC were also discovered (Additional file 6: Figure S5, Additional file 7: Figure S6). 9 from 19 exosome-based proteins specific to LM within NSCLC exhibited strong diagnostic efficacy, while combinations consisting of such nine proteins performed synergistically when compared to individual proteins, having AUC = 0.986 (Additional file 6: Figure S5B-D). FCGR2A, APOH, STX7, and ICAM3 had the best diagnostic capacity within LM within SCLC diagnostic model (Additional file 7: Figure S6C), and their combinations had the highest AUC of 0.961 (Additional file 7: Figure S6D).

### The expression levels and diagnostic role of plasma exosomal proteins validated in an independent cohort

To further confirm the diagnostic value of plasma-derived exosomal proteins in LC patients with LM or BM, we included 110 LC patients and 25 HC as the validation cohort. The validation cohort consisted of 40 LC patients with solitary LM, 32 LC patients with solitary BM and 38 LA, and 25 HC. Table 2 showed the clinicopathological characteristics of these individuals.

**Fig. 11:**
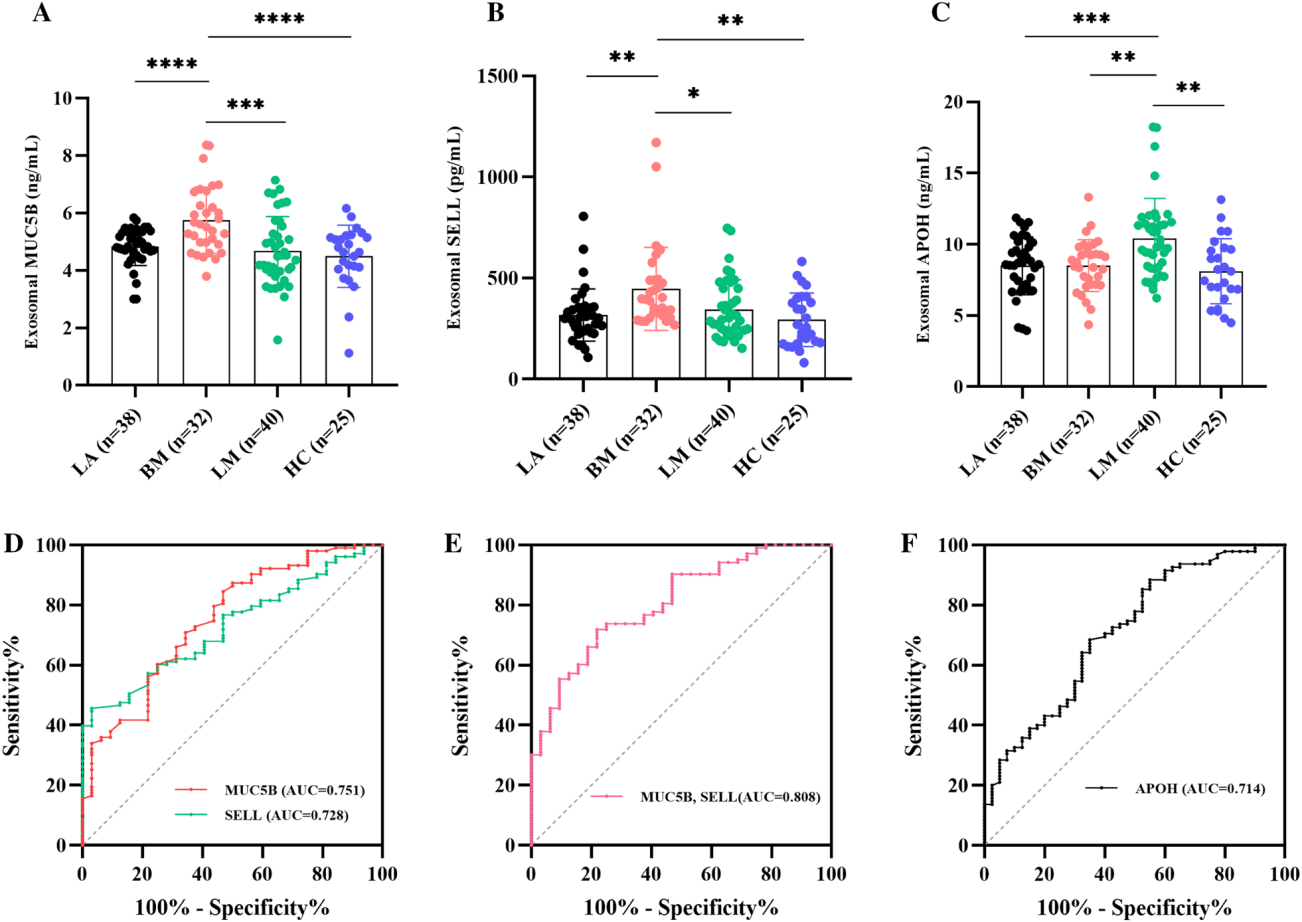
The expression levels and diagnostic role of plasma exosomal proteins validated in lung cancer patients and healthy controls. **A**, **B** The expression levels of plasma exosomal MUC5B and SELL were significantly elevated in lung cancer patients with BM. **C** The expression levels of plasma exosomal APOH were significantly upregulated in lung cancer patients with LM. **D** ROC curves assessed the diagnostic ability of plasma exosomal MUC5B and SELL in lung cancer patients with BM. **E** Plasma exosomal MUC5B and SELL combinations had the greatest AUC (0.808). **F** ROC curves assessed the diagnostic ability of plasma exosomal APOH in lung cancer patients with LM

We isolated exosomes from plasma samples of 110 LC patients and 25 HC by SEC and detected the expression levels of MUC5B, SELL and APOH in these plasma-derived exosomes using ELISA assay. Plasma exosomal MUC5B expression levels were significantly elevated in LC patients with BM, when compared to HC and LC patients with LM and LA (Fig. 11A). Similarly, compared with HC and LC patients with LM and LA, the expression level of plasma exosomal SELL was significantly up-regulated in LC patients with BM (Fig. 11B). However, there were no significant differences in the expression levels of plasma exosomal MUC5B and SELL in the HC, LA, and LM groups. Furthermore, plasma exosomal APOH levels were significantly higher in LC patients with LM than in HC and LC patients with BM and LA (Fig. 11C), and there were no significant differences in the HC, LA, and BM groups. ROC analyses showed that plasma exosomal MUC5B and SELL could differentiate LC patients with BM from HC and LC patients with LM and LA with an AUC value of 0.751 (95% CI 0.655–0.847, p < 0.001) and 0.728 (95% CI 0.641–0.815, p < 0.001), respectively (Fig. 11D). Moreover, their combinations had the highest AUC of 0.808(95% CI 0.728–0.888, p < 0.001) (Fig. 11E). In addition, plasma exosomal APOH also had a certain diagnostic value in LC patients with LM, with a diagnostic capacity of 0.714 (95% CI 0.618–0.809, p < 0.001) (Fig. 11F). In conclusion, these results demonstrated that plasma exosomal MUC5B and SELL could serve as potential biomarkers for diagnosing LC patients with BM, and plasma exosomal APOH was a novel potential diagnostic biomarker of LC patients with LM.

## Discussion

Exosome proteomics is one of the most intriguing topics in clinical oncology. According to several studies, cancer cells’ exosomes carry several proteins to promote organ-specific metastasis [15, 21, 22]. There are several databases for exosome-based proteins, such as ExoCarta [18, 19] and Vesiclepediab [20], while public data for exosome-based proteins in lung cancer metastasis are still scarce. Exosomes have a lipid bilayer membrane structure, which can effectively protect their contents from degradation by external proteases and other enzymes. Previous studies have shown that exosomal proteins are stable over a long period of storage time, and phosphorylated exosomal proteins could be isolated from samples frozen for 5 years [23]. Therefore, exosomes may act as non-invasive biomarkers with promise for prognosis, diagnosis, and prediction since they are stable in blood [24–27]. In this study, we collected plasma samples from LC cases of solitary liver and brain metastases, LA, and HC within 5 years at our center to assess the LM and BM pathogenesis and find potential novel organ-specific proteomic biomarkers. We effectively separated and purified the plasma exosomes of the four cohorts using SEC, and we then thoroughly characterized these exosomes using TEM, NTA, and WB studies to ensure their purity. We next used LC–MS/MS to do a systemic proteome study on these exosomes.

To our knowledge, this is the first investigation into differently expressed proteins in solitary BM and LM exosomes made from plasma. Exosome-based protein profiles from LC cases of isolated liver and brain metastases were examined to determine their homogeneity and heterogeneity. The LM-DEEPs and BM-DEEPs were specifically engaged in regulating actin cytoskeleton, humoral immunological response, proteomic triggering cascade, binding of cell adhesion molecules, extracellular matrix containing collagen, and ECM-receptor interaction. These findings provided useful examples of the pathological similarities between lung cancers within BM and LM, indicating that proteins from exosomes were primarily released into extracellular matrix by particular cells that acted as ligands activating signaling pathways, and mediating processes like immune response, cell adhesion, and migration. We found that they exhibited considerable heterogeneity in addition to homogeneity, suggesting that exosome-based proteins may play a role in organ-specific targeting and metastasis. For instance, the lipoprotein, integrin protein, proteoglycans, and heat shock protein were linked to liver tropic metastasis, while calcium-dependent protein and exosome-derived S100 protein were strongly linked to brain tropic metastasis.

Also, AHSG, APOC3, F2, HP, and ACTB were simultaneously present within hub LM-DEEPs and the hub BM-DEEPs, suggesting that those exosome-based proteins may play an essential part in start and development of BM and LM. ACTB is a highly conserved cytoskeleton structural protein, and a recent study also showed that ACTB was a novel extracellular vesicle marker [17]. Previous studies found that APOC3, F2, and AHSG were critical factors within LM of colorectal cancer and may be used as novel biomarkers for predicting the LM of colorectal cancer [28]. Of note, studies on exosomes carrying these proteins and tumor metastasis are still limited, and further exploration is needed. Our study first illustrates that these hub exosome-based proteins are related to liver and brain metastasis of LC, which may provide data support for future related studies.

Interestingly, the exosome-based protein landscapes of SCLC and NSCLC with liver or brain metastases varied. It should be noted that the distinctive BM_SCLC_DEEPs were scarce within significant KEGG pathways, resulting from the SCLC within BM sample size being too small. In GO analysis, the distinctive BM_NSCLC_DEEPs were mostly connected with structural components of extracellular matrix, integrin/glycosaminoglycan binding, while distinctive BM_SCLC_DEEPs were primarily abundant within cholesterol binding, cellular/tissue-based homeostasis, together with cell adhesion. This investigation discovered special LM_NSCLC_DEEPs were primarily engaged in developing the actin cytoskeleton, cholesterol metabolism, and the apelin signaling pathway. The distinctive LM_SCLC_DEEPs, however, were primarily abundant within immune system-related pathways, the development of extracellular matrix, and binding of integrin, calcium-dependent protein, and S100 protein binding. The aforementioned data revealed variability within plasma-stemming exosome-based proteome for SCLC/NSCLC cases, partially explaining varying metastasis traits within SCLC/NSCLC cases.

Furthermore, our work supports the hypothesis that exosome-based proteins could be employed as a liquid biopsy tool to help diagnose and guide therapeutic strategies for treating cancer metastasis, which is clinically valuable and provides data support for future prospective studies. For instance, based on the soft clustering method and nested comparisons, we found that MUC5B and SELL have potential diagnostic values for lung cancer cases of BM, and the best accuracy was achieved when separating cases of BM and LA from LM using the combination of APOH, CD81, and CCT5. Subsequently, to further validate the expression levels and the diagnostic performance of the exosomal protein markers identified in this study, including MUC5B, SELL and APOH, we included 110 LC patients and 25 HC as a validation cohort. The results further demonstrated that plasma exosomal MUC5B and SELL could serve as potential biomarkers for diagnosing LC patients with BM, and plasma exosomal APOH could be a novel potential diagnostic biomarker for LC patients with LM.

MUC5B is a high-molecular glycoprotein secreted by the bronchial glands and is required for airway defense and controlling bacterial infection [29]. Previous studies have shown that MUC5B is closely associated with tumor metastasis. For example, MUC5B expression is related to the aggressive behavior of breast cancer cells [30], and when the expression of MUC5B was silenced, the proliferation, clonogenicity, and adhesion ability of the breast cancer cells were decreased [31]. Yuan et al. reported that MUC5B-AS1 could increase MUC5B expression by forming an RNA-RNA duplex with MUC5B, thus promoting the migration, invasion and metastasis of lung cancer cells. Moreover, the high expression of MUC5B was significantly related to poor outcomes in lung adenocarcinoma [32] and may increase the likelihood of post-operative lung cancer recurrence or metastases [33]. Besides, some previously published research claimed that exosomes from saliva or serum that included MUC5B might be utilized to identify lung cancer [34, 35]. As a result, based on previous studies and this study, we hypothesized that MUC5B plays an important role in tumor metastasis, and exosome-based MUC5B would be significant in the incidence and progression of lung cancer as well as the catalyst for brain metastasis. SELL is a key vascular adhesion molecule. Qian et al. demonstrated that SELL could promote the homing of tumor cells to lymph nodes in a transgenic mouse model, thus leading to tumor metastasis [36]. Chen et al. further found that SELL-mediated NK cell recruitment plays a crucial role in the control of tumor metastasis into lymph nodes [37]. Moreover, SELL-dependent recruitment of monocytes can directly increase the transendothelial migration and vascular permeability of cancer cells [38]. This evidence confirms the potential role and mechanism of SELL in cancer metastasis. APOH belongs to the apolipoprotein family, and its biological functions are primarily involved in the cholesterol transport and lipoprotein metabolism process. Recently, several studies found that APOH is a key hub gene in colorectal cancer liver metastasis [28], and colorectal cancer patients with metastasis with higher levels of APOH expression also had worse prognoses and survival [39]. Moreover, APOH can bind to lipopolysaccharide and activate NF-KB through TLR4 pathway, thus affecting the metastasis and invasion of tumor cells [39], which may be one of the potential mechanism of its involvement in tumor metastasis. However, the role of exosomal MUC5B, SELL and APOH in tumor metastasis has not been reported, and the mechanism of their involvement in tumor metastasis remains to be further explored. Additionally, we investigated the diagnostic indicators of LM and BM in different pathological forms of LC, offering some theoretical insight into precise grading and management of lung cancer. For instance, CD9 may be a novel biomarker in SCLC, whereas SELL, PODXL, and DSG1 may be potential biomarkers in NSCLC for the BM diagnosis.

These results set the groundwork for future research into molecular processes and biomarkers connected to LM and BM in LC patients as well as some unique prospects for the exosome proteomic analysis of lung cancer. More importantly, our study provides novel insights for developing new and efficacious therapeutics against LC with liver or brain metastasis, which may be highly relevant for the clinical management of lung cancer patients. This investigation does have limitations. Firstly, to confirm the findings of this study, plasma samples from early-stage lung cancer patients are required. Secondly, some prospective studies are needed to further confirm the conclusions and clinical significance of this study. Thirdly, the functional role of the above DEEPs in metastasis needs to be evaluated in greater depth using in vitro and in vivo models.

In conclusion, the plasma-stemming exosomes’ proteome profiles from LC cases of liver and brain metastasis were shown to vary. Additionally, the exosome-based protein landscape varied across distinct clinical kinds of LC with liver or brain metastasis. Within the present study, we also put forward a few prospective biomarkers and speculative molecular pathways that may offer valuable therapeutic and diagnostic insights for future research on LC metastasis for liver and brain.

## Methods

### Plasma samples

This research included 42 cases of metastatic LC (including 16 solitary LM and 26 solitary BM), 25 LA, and 5 HC. Table 1 displayed the clinical characteristics of the individuals. Another cohort of 110 cases of LC (including 40 solitary LM, 32 solitary BM and 38 LA) and 25 HC were included to verify the expression levels and the diagnostic performance of the exosomal protein markers identified in this study. The characteristics of these individuals in the validation cohort were shown in Table 2. Patients who were at least 18 years of age were eligible for this study if they had imaging and pathologically confirmed lung cancer without organ metastasis, lung cancer with solitary brain metastasis and lung cancer with solitary liver metastasis. All patients had not received any treatment. Patients were excluded if they had both brain metastasis and liver metastasis. Patients were also excluded if they had received any therapies, or had a history of cancer, infectious disease, or active autoimmune disease. All of the plasma samples were from our center within 5 years. Centrifugation was used to separate the plasma (2000*g*/20 min/4 °C). After centrifugation, lipemia- and hemolysis-free supernatant was kept at − 80 °C until exosome separation could be performed. The research was authorized by the ethics committees of the Peking Union Medical College and the National Cancer Center/National Clinical Research Center for Cancer/Cancer Hospital, Chinese Academy of Medical Sciences.

**Table 1:** Baseline patient characteristics

Characteristics	Brain metastasis (N = 26)	Liver metastasis (N = 16)	Locally advanced (N = 25)	Healthy control (N = 5)
Age group-no. (%)				
< 55 year	6 (23.1%)	3 (18.8%)	2 (8.0%)	5 (100.0%)
55–64 year	14 (53.8%)	8 (50.0%)	15 (60.0%)	0 (0.0%)
≥ 65 year	6 (23.1%)	5 (31.2%)	8(32.0%)	0 (0.0%)
Median age (range)-year	59 (40–77)	61 (41–70)	61 (44–72)	33 (25–46)
Gender				
Male-no. (%)	17 (65.4%)	11 (68.8%)	19 (76.0%)	1 (20.0%)
Female-no. (%)	9 (34.6%)	5 (31.2%)	6 (24.0%)	4 (80.0%)
History of tobacco use-no. (%)				
Never	9 (34.6%)	3 (18.8%)	10 (40.0%)	5 (100.0%)
Previous and current	15 (57.7%)	10 (62.4%)	14 (56.0%)	0 (0.0%)
Unknown	2 (7.7%)	3 (18.8%)	1 (4.0%)	0 (0.0%)
Histologic subtype-no. (%)				
Non-small cell lung cancer	20 (76.9%)	8 (50.0%)	15 (60.0%)	–
Small cell lung cancer	6 (23.1%)	8 (50.0%)	10 (40.0%)	–

**Table 2:** Characteristics of 135 individuals in the validation cohort

Characteristics	Brain metastasis (N = 32)	Liver metastasis (N = 40)	Locally advanced (N = 38)	Healthy controls (N = 25)
Age group-no. (%)				
< 55 year	7 (21.9%)	14 (35.0%)	6 (15.8%)	7 (28.0%)
55–64 year	19 (59.4%)	13 (32.5%)	14 (36.8%)	10 (40.0%)
≥ 65 year	6 (18.7%)	13 (32.5%)	18(47.4%)	8 (32.0%)
Median age (range)-year	59 (35–78)	59 (42–78)	63 (45–78)	58 (30–79)
Gender				
Male-no. (%)	21 (65.6%)	27 (67.5%)	27 (71.1%)	17 (68.0%)
Female-no. (%)	11 (34.4%)	13 (32.5%)	11 (28.9%)	8 (32.0%)
History of tobacco use-no. (%)				
Never	15 (46.9%)	17 (42.5%)	11 (28.9%)	10 (40.0%)
Previous and current	15 (46.9%)	21 (52.5%)	26 (68.4%)	14 (56.0%)
Unknown	2 (6.2%)	2 (5.0%)	1 (2.7%)	1 (4.0%)
Histologic subtype-no. (%)				
Non-small cell lung cancer	22 (68.8%)	25 (62.5%)	22 (57.9%)	–
Small cell lung cancer	10 (31.2%)	15 (37.5%)	16 (42.1%)	–

### Isolation of the exosomes

The plasma samples were processed for exosome separation by size exclusion chromatography (qEVoriginal-5® Pack IZON Science™, USA). After centrifuging the 1 ml plasma (1500*g*/10 min; 10,000*g*/30 min/4 °C), supernatants were kept on-ice until required. After 15 ml PBS was used to clean the columns and equilibrate them, 500 ul of plasma sample was added for columns. 1.5 ml of elute was harvested for analyzing exosomes after discarding the first 3 ml (void volume). After exosome collection, separation columns were washed using 20 ml PBS, 10 ml 0.5 M NaOH, and 20 ml PBS to equilibrate. Then, the next sample was loaded. Notably, PBS used in this experiment was filtered through a 0.22 μm filtration unit (Merck Millipore, USA), and the loading volume for each sample was 500 ul. Each column was used twice. After collecting samples, we isolated exosomes by centrifuging them at 7500*g* for 30 min through a molecular weight cut-off (MWCO) membrane of 10 kDa (Merck Millipore, USA) using an Amicon Ultra-4 centrifugal filter. After exosomes were isolated, they were frozen at − 80 °C for further analysis.

### Transmission electron microscope (TEM)

Under the TEM, the exosome-based morphology was examined. PBS was used to dilute the purified exosomes ten times before being applied to a copper grid for 1 min. Any surplus solution was then wiped away using filter paper. The phosphotungstic acid solution was used to negatively stain grid harboring absorbed exosomes for 1 min. The dried grid was then looked at using TEM (JEOL®, JEM-1400 120 kV, Japan) after that.

### Nanoparticle tracking analysis (NTA)

The NTA platform (Nanosight NS300®, Malvern Instruments™, UK) evaluated 100 times PBS diluted exosomes in accordance with the established technique. Each sample underwent at least three analyses.

### Western blotting (WB)

The usual methodology for Western blotting was followed. A PVDF membrane was used to receive the 30 g of total protein that was extracted from exosomes and placed onto a 15% SDS-PAGE gel. 5% skimmed-milk + TBS-T blocked the membranes (60 min). Consequently, membranes were exposed to goat anti-rabbit IgG-HRP secondary antibody (1: 2500 dil., Abcam™, UK) for 120 min at room temperature after first incubating with primary antibodies against CD81 and CD9 (1:1000 dil., CST™, USA), 4 °C/overnight. The membranes were then shaken and rinsed three times for 15 min with TBS-T before being imaged through Chemiluminescent Substrate System® (Thermo Scientific™, USA).

### Collection/breakdown for exosome-based proteins

In order for extracting within isolated exosomes, lysis was performed in RIPA lysis buffer at 4 °C for 40 min in a 1:1 ratio. Exosome-based proteomic content was determined through BCA assay® kit (Thermo Scientific™, USA). Each sample’s 30 ug of isolated exosome-based proteins was then prepared using the Filter-Aided Sample Preparation (FASP) method. In a nutshell, 8 M urea (pH = 8.5) was combined with proteins within Vivacon 500® filtering platform (Sartorius Stedim Biotech™) before centrifuging the mixture twice (2 × 14,000*g*/15 min/20 °C). Samples were then incubated (37 °C/1 h) while 8 M urea/0.05 M Tris-(2-carboxyethyl)-phosphine (TCEP) were introduced. Samples were then treated with 0.1 M iodoacetamide (IAA), and incubated (37 °C/60 min/darkness; 14,000*g*/15 min/20 °C). Twice, 50 mM ammonium bicarbonate was used to wash the ultrafiltration tube. Finally, each ultrafiltration tube received trypsin containing ammonium bicarbonate solution (50 mM) before incubating overnight (37 °C). Peptides were then recovered through centrifuging for LC–MS/MS assessments.

### Liquid chromatography-MS/MS analysis

A Q-Exactive HF mass spectrometer (MS) (Thermo Scientific, USA) was used within LC–MS/MS study. Thermo Scientific, USA’s precolumn was used to enrich and desalt 5 ul of the peptide before separating it at a 300 ml/min flow rate for 120 min through the C18 column having 2–40% gradient mobile phase B. The main mass spectrometer’s resolution was 60,000, and its scanning range was 300–1800 m/z. The MS was run within top-ranking 20 (data-dependent acquisition method) mode. Twenty parent-ions having greatest signals were chosen for secondary mass spectrometry scans after finishing the first mass spectrometry. The dynamic exclusion was set at 30 s, the resolution was 15,000, and the fragmentation mode was HCD. MaxQuant (v1.4.1.2, http://www.maxquant.org/) searched each sample raw data file and identify and quantify any observable characteristics.

### ELISA assay

Human MUC5B ELISA Kit (Tianjin Anoric Bio-technology, China), Human SELL ELISA Kit (Tianjin Anoric Bio-technology, China) and Human APOH ELISA Kit (Tianjin Anoric Bio-technology, China) were used to evaluate the abundance of MUC5B, SELL and APOH in plasma-derived exosomes of 110 LC patients and 25 HC in the validation cohort, respectively, according to the manufacturer’s instructions. Total protein content was normalized by BCA assay.

### Bioinformatics analyses

Label-Free Quantification (LFQ) intensity was used to quantify the proteins, and those lacking both LFQ intensity and MS/MS counts were eliminated. For subsequent analysis, LFQ intensities were adjusted through median in proteins registering LFQ intensity ≠ 0 within a minimum of 1/72 overall-samples. ExoCarta (http://www.exocarta.org) and Vesiclepedia (http://www.microvesicles.org) databases were compared with the discovered exosome-based proteins. GO enrichment analysis and KEGG pathway assessments were conducted through “ClusterProfiler” R package (http://www.bioconductor.org). Venn diagram was constructed through two online resources (http://bioinformatics.psb.ugent.be/webtools/Venn/) and https://bioinfogp.cnb.csic.es/tools/venny/index.html). DEEPs were filtered using the R “limma” package, and heat-mapping/soft-clustering were produced using “Pheatmap” and “Mfuzz” tools, respectively. Complete networks were shown in Cytoscape after being analyzed with the help of STRING repository (http://string-db.org) for PPI having average scorings > 0.7. The hub proteins were screened using Cytoscape’s CytoHubba plug-in, with such co-expression networking evaluated using the GeneMANIA database (http://www.genemania.org/). These six methods generally consist of MNC, MCC, Degree, Closeness, EPC, and Radiality. ROC curves and AUC assessed diagnosis efficacy for possible DEEPs.

### Statistical analyses

Datasets were assessed through SPSS® 22.0 (SPSS Inc.™, USA) together with GraphPad® (GraphPad Software Inc.™, USA). Student t-test was applied to compare the expression of exosomal MUC5B, SELL and APOH between HC, BM, LM and LA. The ‘limma’ tool from Bioconductor assessed variations across cohorts, with p < 0.05 conferring statistical significance.

### Supplementary Information

**Additional file 1: Table S1.** Proteins identified by proteomic analysis using LFQ intensity value ≠ 0 in at least one out of 72 samples. **Table S2.** Differentially expressed exosomal proteins in lung cancer patients with brain meatstasis vs. locally advanced comparison (fold change ≥ 2 or ≤ 0.5 and p < 0.05). **Table S3.** Differentially expressed exosomal proteins in lung cancer patients with liver meatstasis vs. locally advanced comparison (fold change ≥ 2 or ≤ 0.5 and p < 0.05). **Table S4.** Exosomal proteins were clustered by soft clustering in HC, LA and BM cohorts. **Table S5.** Differentially expressed exosomal proteins in lung cancer patients with brain meatstasis vs. liver metastasis comparison (fold change ≥ 2 or ≤ 0.5 and p < 0.05). **Table S6.** Exosomal proteins were clustered by soft clustering in HC, LA and LM cohorts.

**Additional file 2: Figure S1.** Proteomic profiling for plasma-stemming exosomes. (A) Venn diagram showed the exosome-based proteins identified in this investigation compared to documented vesicular top-ranking 100 proteins within Exocarta/Vesiclepedia repositories. (B) The biological processes (BP) and molecular functions (MF) of GO analysis of plasma-stemming exosomes.

**Additional file 3: Figure S2.** KEGG pathway analysis of plasma-stemming exosomes.

**Additional file 4: Figure S3.** Diagnostic utility for the selected plasma-stemming exosome-based proteins in NSCLC patients with BM. (A) 10 soft clusters of BM in NSCLC. The horizontal axis indicates the initiation and progression of BM in NSCLC. The vertical axis represents expression changes of exosomal proteins. (B) Nested analyses of NSCLC_BM versus NSCLC_LM and NSCLC_BM versus NSCLC_LA. ACTB, SELL, DSG1 and PODXL were in cluster A. (C) ROC curve analysis for distinguishing NSCLC patients with BM from LM and LA. (D) The area under the ROC curve of the three exosomal proteins combinations.

**Additional file 5: Figure S4.** Diagnostic utility for the selected plasma-stemming exosome-based proteins in SCLC patients with BM. (A) 10 soft clusters of BM in SCLC. The horizontal axis indicates the initiation and progression of BM in SCLC. The vertical axis represents expression changes of exosomal proteins. (B) Nested analyses of SCLC_BM versus SCLC_LM and SCLC_BM versus SCLC_LA. Only CD9 was in cluster A. (C) The area under the ROC curve of CD9.

**Additional file 6: Figure S5.** Diagnostic utility for the selected plasma-stemming exosome-based proteins in NSCLC patients with LM. (A) 10 soft clusters of LM in NSCLC. The horizontal axis indicates the initiation and progression of LM in NSCLC. The vertical axis represents expression changes of exosomal proteins. (B) Nested analyses of NSCLC_LM versus NSCLC_LA and NSCLC_LM versus NSCLC_BM. 9 proteins belonged to the overlapping proteins of NSCLC_LM versus NSCLC_LA and NSCLC_LM versus NSCLC_BM, as well as to cluster A or B proteins. (C) ROC curve analysis for distinguishing NSCLC patients with LM from BM and LA. (D) The 9 exosomal proteins combinations had the greatest AUC (0.986).

**Additional file 7: Figure S6.** Diagnostic utility for the selected plasma-stemming exosome-based proteins in SCLC patients with LM. (A) 10 soft clusters of LM in SCLC. The horizontal axis indicates the initiation and progression of LM in SCLC. The vertical axis represents expression changes of exosomal proteins. (B) Nested analyses of SCLC_LM versus SCLC_LA and SCLC_LM versus SCLC_BM. 4 proteins belonged to the overlapping proteins of SCLC_LM versus SCLC_LA and SCLC_LM versus SCLC_BM, as well as to cluster A or B proteins. (C) ROC curve analysis to distinguish SCLC patients with LM from BM and LA. (D) The 4 exosomal proteins combinations had the greatest AUC (0.961).

## Data Availability

All data generated or analyzed in this study are included in this article. Other data that are relevant to this article are available from the corresponding author upon reasonable request.

## Abbreviations

BM: Brain metastasis
LM: Liver metastasis
LA: Local advanced
HC: Healthy control
NSCLC: Non-small cell lung cancer
SCLC: Small cell lung cancer
TEM: Transmission electron microscope
NTA: Nanoparticle tracking analysis
WB: Western blotting
BM-DEEPs: Brain metastases-linked differentially expressed exosome-based proteins
LM-DEEPs: Liver metastases-linked differentially expressed exosome-based proteins
GO: Gene Ontology
KEGG: Kyoto Encyclopedia of Genes and Genomes
CC: Cellular component
MF: Molecular function
BP: Biological process
BM_NSCLC_DEEPs: Differentially expressed exosome-based proteins associated with BM in NSCLC
BM_SCLC_DEEPs: Differentially expressed exosome-based proteins associated with BM in SCLC
LM_NSCLC_DEEPs: Differentially expressed exosome-based proteins associated with LM in NSCLC
LM_SCLC_DEEPs: Differentially expressed exosome-based proteins associated with LM in SCLC
