# Supplementary figures and images for: Comparative proteomic profiling of plasma exosomes in lung cancer cases of liver and brain metastasis
Source: Cell Biosci. 2023 Sep 28;13:180. doi: 10.1186/s13578-023-01112-5 (PMC10540327; doi:10.1186/s13578-023-01112-5)

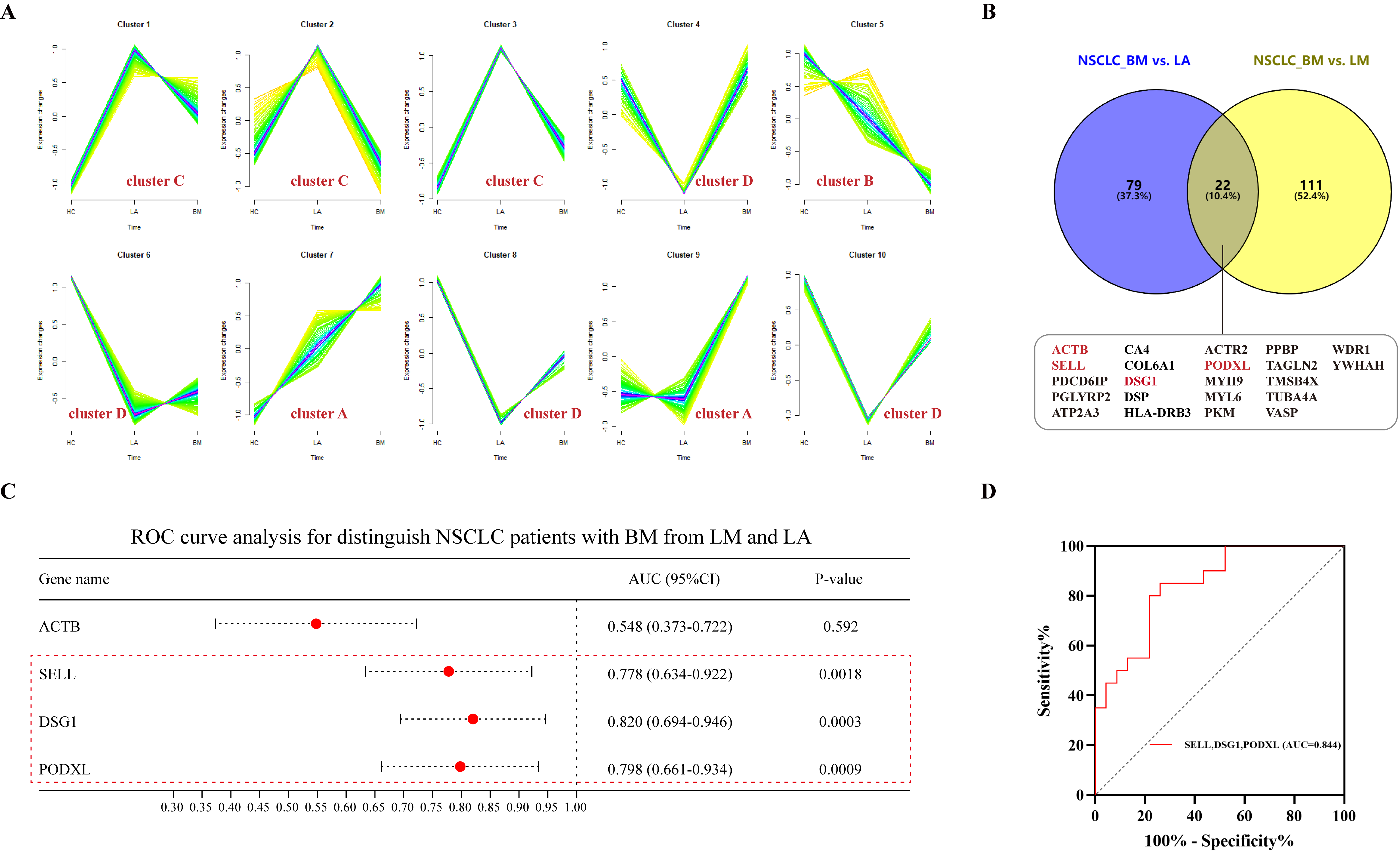

Supplement: Supplementary file 4 — Additional file 4: Figure S3. Diagnostic utility for the selected plasma-stemming exosome-based proteins in NSCLC patients with BM. (A) 10 soft clusters of BM in NSCLC. The horizontal axis indicates the initiation and progression of BM in NSCLC. The vertical axis represents expression changes of exosomal proteins. (B) Nested analyses of NSCLC_BM versus NSCLC_LM and NSCLC_BM versus NSCLC_LA. ACTB, SELL, DSG1 and PODXL were in cluster A. (C) ROC curve analysis for distinguishing NSCLC patients with BM from LM and LA. (D) The area under the ROC curve of the three exosomal proteins combinations. [file 13578_2023_1112_MOESM4_ESM.tif]

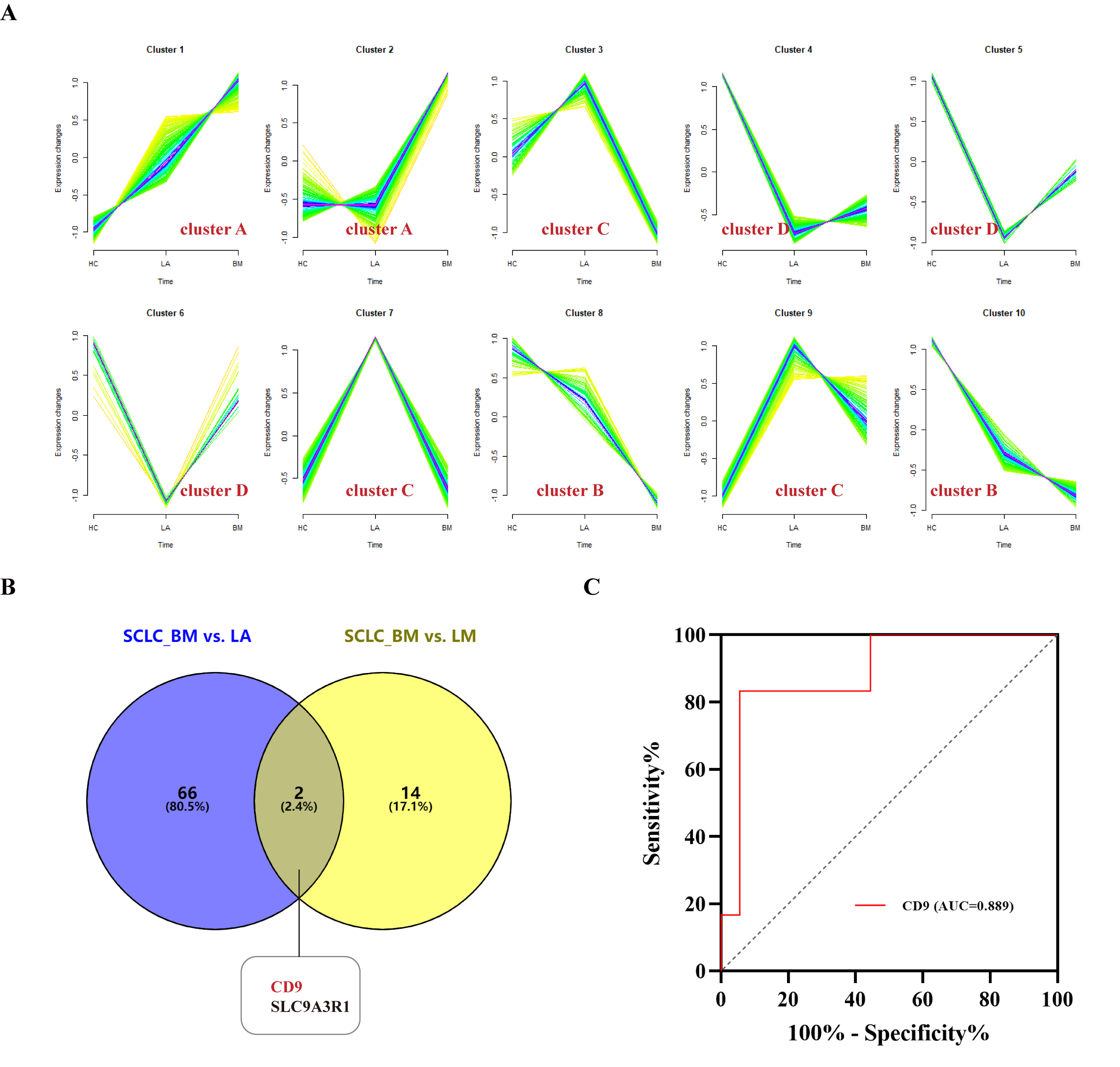

Supplement: Supplementary file 5 — Additional file 5: Figure S4. Diagnostic utility for the selected plasma-stemming exosome-based proteins in SCLC patients with BM. (A) 10 soft clusters of BM in SCLC. The horizontal axis indicates the initiation and progression of BM in SCLC. The vertical axis represents expression changes of exosomal proteins. (B) Nested analyses of SCLC_BM versus SCLC_LM and SCLC_BM versus SCLC_LA. Only CD9 was in cluster A. (C) The area under the ROC curve of CD9. [file 13578_2023_1112_MOESM5_ESM.tif]
